# Drug-Resistant Temporal Lobe Epilepsy Alters the Expression and Functional Coupling to Gαi/o Proteins of CB1 and CB2 Receptors in the Microvasculature of the Human Brain

**DOI:** 10.3389/fnbeh.2020.611780

**Published:** 2021-01-20

**Authors:** María de los Ángeles Nuñez-Lumbreras, José Luis Castañeda-Cabral, María Guadalupe Valle-Dorado, Vicente Sánchez-Valle, Sandra Orozco-Suárez, Rosalinda Guevara-Guzmán, Iris Martínez-Juárez, Mario Alonso-Vanegas, Fruzsina Walter, Maria A. Deli, Francia Carmona-Cruz, Luisa Rocha

**Affiliations:** ^1^Departamento de Farmacobiología, Centro de Investigación y de Estudios Avanzados, Mexico City, Mexico; ^2^Departamento de Farmacología, Centro de Investigación y de Estudios Avanzados, Mexico City, Mexico; ^3^Unidad de Investigación Médica en Enfermedades Neurológicas, Hospital de Especialidades, Centro Médico Nacional Siglo XXI, Instituto Mexicano del Seguro Social, Mexico City, Mexico; ^4^Departamento de Fisiología, Facultad de Medicina, Universidad Nacional Autónoma de México, Mexico City, Mexico; ^5^Instituto Nacional de Neurología y Neurocirugía Manuel Velasco Suárez (INNNMVS), Mexico City, Mexico; ^6^Centro Internacional de Cirugía de Epilepsia, Hospital HMG-Coyoacán, Mexico City, Mexico; ^7^Institute of Biophysics, Biological Research Centre, Hungarian Academy of Sciences, Szeged, Hungary

**Keywords:** mesial temporal lobe epilepsy, drug-resistance, blood-brain barrier, endocannabinoid system, CB1 receptors, CB2 receptors

## Abstract

Cannabinoid receptors 1 and 2 (CB1 and CB2, respectively) play an important role in maintaining the integrity of the blood–brain barrier (BBB). On the other hand, BBB dysfunction is a common feature in drug-resistant epilepsy. The focus of the present study was to characterize protein expression levels and Gαi/o protein-induced activation by CB1 and CB2 receptors in the microvascular endothelial cells (MECs) isolated from the brain of patients with drug-resistant mesial temporal lobe epilepsy (DR-MTLE). MECs were isolated from the hippocampus and temporal neocortex of 12 patients with DR-MTLE and 12 non-epileptic autopsies. Immunofluorescence experiments were carried out to determine the localization of CB1 and CB2 receptors in the different cell elements of MECs. Protein expression levels of CB1 and CB2 receptors were determined by Western blot experiments. [^35^S]-GTPγS binding assay was used to evaluate the Gαi/o protein activation induced by specific agonists. Immunofluorescent double-labeling showed that CB1 and CB2 receptors colocalize with tight junction proteins (claudin-5, occludin, and zonula occludens-1), glial fibrillary acidic protein and platelet-derived growth factor receptor-β. These results support that CB1 and CB2 receptors are expressed in the human isolated microvessels fragments consisting of MECs, astrocyte end feet, and pericytes. The hippocampal microvasculature of patients with DR-MTLE presented lower protein expression of CB1 and CB2 receptors (66 and 43%, respectively; *p* < 0.001). However, its Gαi/o protein activation was with high efficiency (CB1, 251%, *p* < 0.0008; CB2, 255%, *p* < 0.0001). Microvasculature of temporal neocortex presented protein overexpression of CB1 and CB2 receptors (35 and 41%, respectively; *p* < 0.01). Their coupled Gαi/o protein activation was with higher efficiency for CB1 receptors (103%, *p* < 0.006), but lower potency (*p* < 0.004) for CB2 receptors. The present study revealed opposite changes in the protein expression of CB1 and CB2 receptors when hippocampus (diminished expression of CB1 and CB2) and temporal neocortex (increased expression of CB1 and CB2) were compared. However, the exposure to specific CB1 and CB2 agonists results in high efficiency for activation of coupled Gαi/o proteins in the brain microvasculature of patients with DR-MTLE. CB1 and CB2 receptors with high efficiency could represent a therapeutic target to maintain the integrity of the BBB in patients with DR-MTLE.

## Introduction

The blood–brain barrier (BBB) regulates the access of molecules, drugs, neurotoxins, and pathogens from the bloodstream into the cerebral parenchyma. The close interaction between microvascular endothelial cells (MECs), the main components of the BBB, and other elements of the neurovascular unit (astrocytes, pericytes, neurons, and basement membrane) plays a significant role in the homeostasis of the central nervous system (Stanimirovic and Friedman, 2012). MECs present tight junctions, which participate in the control of transcytosis. Tight junctions involve the interaction of transmembrane (occludin, claudins, and junctional adhesion molecules), as well as cytoplasmic [zonula occludens (ZO)] proteins (Hawkins and Davis, 2005). Changes in the expression and interaction of these proteins facilitate BBB disruption (Kook et al., 2012).

Studies indicate that cannabinoid receptors 1 and 2 (CB1 and CB2, respectively) are expressed in different components of the BBB (Molina-Holgado et al., 2002; Golech et al., 2004; Zong et al., 2017). The activation of CB1 receptors prevents downregulation of ZO-1, claudin-5, and junctional adhesion molecules-1 proteins and the consequent BBB breakdown in cocultures of human brain MECs and astrocytes (Lu et al., 2008). In turn, the activation of CB2 receptors increases the transendothelial electrical resistance and the expression of tight junction proteins in MECs and reduces neuroinflammation effects (Ramirez et al., 2012). In autopsy brain samples from patients with multiple sclerosis, the BBB overexpressed CB2 receptors in chronic inactive plaques (Zhang et al., 2011), a finding that supports their protective role.

Epilepsy, a neurological disorder characterized by a permanent predisposition to spontaneous and recurrent seizures (Fisher, 2015), is associated with BBB dysfunction (van Vliet et al., 2014; Broekaart et al., 2018). Recent studies from our research group have shown that the neocortical microvasculature from patients with drug-resistant mesial temporal lobe epilepsy (DR-MTLE) exhibited lower protein expression levels of occludin and ZO-1 as compared to control tissues obtained from autopsies, whereas the protein expression levels of the vascular endothelial growth factors (VEGF-A, VEGFR-2) and claudin-5 were increased (Castañeda-Cabral et al., 2020a). Furthermore, the protein expression levels of the proinflammatory cytokines interleukin 1β and tumor necrosis factor α (TNF-α) and its receptor TNF-R1, as well as the nitric oxide synthase, a marker of oxidative stress, were increased in the microvasculature of DR-MTLE patients (Castañeda-Cabral et al., 2020b). These changes suggest important alterations in the integrity of the BBB that may contribute to the pathogenesis of epilepsy.

Considering that CB1 and CB2 receptors play an important role in the integrity of BBB, it is proposed that epilepsy modifies protein expression levels and/or signal transduction pathways mediated by these receptors in the microvasculature of the human brain. Experiments were designed to evaluate protein expression levels and signaling of CB1 and CB2 receptors in the microvasculature of the hippocampus (epileptogenic area) and temporal neocortex (the brain area involved in the spread of seizure activity) of patients with DR-MTLE. The influence of clinical conditions was investigated through correlation analysis.

## Materials and Methods

### Subjects

Brain tissue was obtained from adult patients with DR-MTLE (*n* = 12) who underwent surgery through the Epilepsy Surgery Program of the National Institute of Neurology and Neurosurgery “Manuel Velasco Suárez” (INNNMVS) in Mexico City. All the neurosurgeries were carried out by the same neurosurgeon (MAV). Patients with DR-MTLE showed the following clinical data: age of subjects, 30.2 ± 2.5 years; age at seizure onset, 10.0 ± 2.3 years; epilepsy duration, 20.8 ± 3.3 years; and frequency of seizures, 7.9 ± 1.6 per month (Table 1). Preoperative evaluation for each patient included video electroencephalogram, magnetic resonance imaging, and single-photon emission computed tomography. Subsequently, patients underwent an anterior lobectomy ipsilateral to the epileptic focus. During the surgical procedure, hippocampal and temporal neocortex biopsies were collected immediately after resection, frozen on dry ice, and stored at −70°C until processed. The scientific and ethics committees from INNNMVS and Cinvestav approved the protocol for this experimental study (reference numbers of approval: 101/18 and 055/2018, respectively). Written informed consent was obtained from all participants.

**Table 1:** Clinical data of patients with drug-resistance mesial temporal lobe epilepsy.

**Patient**	**Age (years)**	**Gender**	**Age at seizure onset (years)**	**Epilepsy duration (years)**	**Frequency of seizures**	**ASD**
					**(per month)**	**treatment**
						**before surgery**
416	18	F	9	9	4	CBZ, TPM, CNZ, LVT
428	45	M	8	37	3	PHE, VPA, CBZ, OXC, LMG, TPM, CNZ
429	34	M	1	33	10	PHE, CBZ, TPM, CNZ, CLB, LCS
434	32	F	3	29	4	PHE, OXC, LMG, TPM, LVT
454	26	F	2	26	5	CBZ, OXC
457	35	F	20	15	4	PHE, VPA, OXC
470	30	F	6	24	4	CBZ, TPM, CNZ, LVT
474	44	F	20	24	12	CBZ, CZP
516	17	F	16	1	16	PHE, LVT
517	25	M	24	1	20	VPA, LVT
534	29	F	6	23	8	CBZ, LVT
545	27	M	5	22	5	VPA, CBZ, OXC, TPM

*ASD, antiseizure drug; CBZ, carbamazepine; CLB, clobazam; CNZ, clonazepam; CZP, clorazepate; F, female; LCS, lacosamide; LMG, lamotrigine; LVT, levetiracetam; M, male; OXC, oxcarbazepine; PHE, phenytoin; TPM, topiramate; VPA, valproic acid*.

Samples from the hippocampus and temporal neocortex obtained from 12 autopsies, died of causes not associated with neurological disorders, were evaluated as controls. The mean age of the autopsy subjects (41.0 ± 4.8 years) was not significantly different from that of the patients with DR-MTLE (*p* > 0.05). Autopsy samples were collected with a postmortem interval of 14.8 ± 3.7 h (Table 2). The samples were frozen immediately after resection and stored at −70°C. Autopsies were performed at the Institute of Forensic Sciences in Mexico City. The quality of autopsies was verified by evaluating the integrity of mRNA of each sample.

**Table 2:** Clinical data of non-epileptic autopsies.

**Autopsy**	**Age**	**Gender**	**Cause of death**	**PMI**
	**(years)**			**(hours)**
A1	29	M	Abdominal trauma	13
A2	29	M	Polytrauma	18
A6	49	F	Asphyxia	18
A7	45	M	Asphyxia	18
A8	73	M	Diabetes complications	15
A9	33	F	Thoracic trauma	12
A11	12	F	Asphyxia	14
A12	40	F	Ballistic trauma	20
A14	45	F	Unknown	10
A16	57	M	Heart attack	18
A17	25	F	Thoracic trauma	18
A18	55	M	Thoracolumbar trauma	15

*F, female; M, male; PMI, postmortem interval*.

### Isolation of Human Brain Microvessels

Brain microvessels were obtained from frozen human brain tissue according to a modified version of the protocol described previously by Veszelka et al. (2007) (for further details, see Castañeda-Cabral et al., 2020a). Using this procedure, we previously have found that the isolated microvessels from frozen brain tissue contain MECs, pericytes, and astrocytes end feet (Castañeda-Cabral et al., 2020a). All these cells are also a source of CB1 and CB2 receptors (Benyó et al., 2016).

Briefly, brain tissue was homogenized in Ringer HEPES (RH) buffer solution (150 mM NaCl, 2.2 mM CaCl_2_, 0.2 mM MgCl_2_, 5.2 mM KCl, 2.8 mM glucose, 5 mM HEPES, 6 mM NaHCO_3_, pH 7.4) in a proportion of 4 mL per gram of tissue (4 mL/g of tissue). The homogenates were centrifuged at 1,000 × *g* for 15 min, at 4°C. The obtained pellet was resuspended in 17.5% RH-dextran and then centrifuged at 1,500 × *g* for 15 min, at 4°C. The resulting pellet was resuspended in 2 mL of 1% RH–bovine serum albumin (BSA), whereas the supernatant was centrifuged twice under the same conditions until obtaining three pellets. All the pellets were filtered with a 100-μm mesh. The obtained supernatant was filtered again with a 40-μm mesh. The filtrate was centrifuged at 1,000 × *g*, for 10 min, at 4°C. Subsequently, the pellet was collected in an Eppendorf tube. A sample of each pellet was analyzed using an inverted microscope (Leica DMi1) to evaluate the characteristics of the isolated microvessels. Subsequently, the expression of CD34, a valuable marker for the identification and characterization of capillary endothelial cells, was analyzed in MECs by immunofluorescence experiments. For this purpose, a fraction of the pellet was mounted on slides previously coated with poly-l-lysine and allowed to dry at room temperature for 24 h. The tissue was fixed with cold acetone for 90 min at 4°C and subsequently permeabilized with phosphate-buffered saline (PBS)−0.25% Triton X-100 for 15 min at 4°C. Blocking was performed using 3% BSA-PBS for 50 min at 4°C. Slides were incubated overnight (4°C) with an anti-CD34 antibody followed by incubation with fluorescence-labeled secondary antibody (Alexa Fluor 488, goat anti–rabbit immunoglobulin G, 1:200) for 2 h at 4°C. Subsequently, slides were washed with PBS and incubated with Hoechst 33342 (1:5,000, cat. H3570, lot 1724829, Thermo Fisher Scientific, USA) for 5 min at room temperature for nuclear staining. Finally, slides were mounted with Vectashield mounting medium for fluorescence (cat. CB-1000; Vector Laboratories Inc., USA) and analyzed by confocal microscopy (Eclipse TE2000 microscope, Nikon, Japan). Images were obtained with a Spot RT digital camera (Diagnostic Instruments, USA).

Once the isolation of microvessels was confirmed, each pellet was divided into three fractions for the following experiments: (a) immunofluorescence analysis, (b) Western blot analysis, and (c) [^35^S]-GTPγS binding assay. The pellets were stored at −70°C until further use.

### Immunofluorescence Staining of CB1 and CB2 Receptors in the Human Brain Microvessels

Immunofluorescence experiments were carried out to localize CB1 and CB2 receptors in the different cell elements of human brain microvessels. Their expression was analyzed alone, as well as their colocalization with glial fibrillary acidic protein (GFAP), platelet-derived growth factor receptor-β (PDGFR-β), and tight junction proteins (claudin-5, occludin, and ZO-1). For this purpose, a fraction of the pellet previously obtained was mounted on slides coated with poly-l-lysine and allowed to dry at room temperature for 24 h. The tissue was fixed with cold acetone for 90 min at 4°C and subsequently permeabilized with PBS-0.25% Triton X-100 for 15 min at 4°C. Blocking was performed using 3% BSA-PBS for 50 min at 4°C. Slides containing microvessels followed an overnight primary antibody incubation at 4°C and were exposed to secondary antibody for 2 h at 4°C. Particulars for each antibody, such as primary and secondary antibody information as well as dilutions, are listed in Supplementary Table 1. Subsequently, slides were washed with PBS and incubated with Hoechst 33342 (1:5,000, cat. H3570, lot 1724829, Thermo Fisher Scientific, USA) for 5 min at room temperature for nuclear staining. Finally, slides were mounted with Vectashield mounting medium for fluorescence (cat. CB-1000, Vector Laboratories Inc., USA) and analyzed by confocal microscopy (Eclipse TE2000 microscope, Nikon, Japan). Images were obtained with a Spot RT digital camera (Diagnostic Instruments, USA). As negative control for specificity of staining, the immunohistochemical assay was carried out with the primary antibody omitted that resulted in the absence of immunolabeling. Also, images of Z-stack (17 planes each μm) were used for protein localization. NIS-Elements v.4.50 and ImageJ v.1.50i software were used for protein colocalization.

### Evaluation of CB1 and CB2 Protein Expression by Western Blot

The pellet was homogenized in RIPA lysis buffer (50 mM Tris–HCl, 150 mM NaCl, 1 mM EDTA, and 0.1% Triton X-100, pH 7.5) and a mixture of protease inhibitors (Complete Roche Diagnostics GmbH, Germany). Protein quantification was performed by the Bradford method (Protein Assay Dye, cat. 5000006, Bio-Rad Laboratories, USA). MEC samples (15 μg/lane) were loaded in Laemmli sample buffer (cat. 1610737, Bio-Rad Laboratories, USA) and separated in 10% sodium dodecyl sulfate (SDS)–polyacrylamide gel electrophoresis gel (85 V for 30 min and 100 V for 2 h) using running buffer (25 mM Tris, 192 mM glycine and 0.1% SDS, pH 8.3; cat. 1610723, Bio-Rad Laboratories, USA). Subsequently, the proteins were transferred onto polyvinylidene difluoride membranes (Immun-blot, cat. 1620264, Bio-Rad Laboratories, USA). After electroblotting, membranes were incubated for 1 h at room temperature with 5% blocking solution (Blot-QuickBlocker, cat. WB57, EMD Millipore, USA) dissolved in TBS-T buffer (20 mM Tris, 500 mM NaCl, 0.1% Tween 20, pH 7.5). Subsequently, membranes were incubated overnight at 4°C with the corresponding primary antibody. Membranes were washed with TBS-T and incubated for 1 h with appropriate horseradish peroxidase–coupled secondary antibody (Supplementary Table 1). Blots were developed using Clarity Western ECL Blotting Substrates (Bio-Rad Laboratories, USA) according to the manufacturer's indications. Scanning of the immunoblots was performed, and the density of bands was estimated using ImageJ v.1.50i software. The chemiluminescent data were normalized using β-actin as a constitutive protein, resulting in a relative ratio expression. Each sample was evaluated by duplicate.

### Evaluation of the Gαi/o Protein Activation Induced by CB1 and CB2 Receptors in Human Microvessels

CB1 and CB2 receptors are composed of seven transmembrane α-helix domains and a C-terminal domain (73 and 59 amino acids, respectively) (Matsuda et al., 1990; Munro et al., 1993), which couple to Gαi/o proteins to induce their cell effects (Abood and Martin, 1996). The interaction of specific agonists with these receptors facilitates the exchange of guanosine triphosphate (GTP) for guanosine diphosphate (GDP) in the Gαi/o subunit. For the present study, a binding assay was used to evaluate the efficacy and potency of specific CB1 and CB2 agonists to increase the binding of [^35^S]-GTPγS, a radiolabeled non-hydrolyzable GTP analog, to Gαi/o proteins.

Cell membranes of microvessels were obtained according to the following procedure. The pellet was resuspended, homogenized in a buffer solution (2 mM Tris–EDTA, 320 mM sucrose, 5 mg/mL BSA, 5 mM MgCl_2_; pH 7.4), and centrifuged three times at 1,000 × *g* for 10 min, at 4°C. The first and second supernatants were collected in a tube and centrifuged at 39,000 × *g* for 30 min at 4°C, whereas the third supernatant was discarded. The resulting pellet was resuspended in a buffer solution (50 mM Tris–HCl, 2 mM Tris–EDTA, 3 mM MgCl_2_, pH 7.4). A fraction of the pellet was used for protein quantification according to the Bradford method (Protein Assay Dye, cat. 5000006, Bio-Rad Laboratories, USA).

Cell membranes were used for binding assays as previously described (Catani and Gasperi, 2016), with some modifications. A total of 8 μg of membrane protein per sample was incubated (60 min, 37°C) in 0.8 mL of buffer solution (50 mM Tris–HCl, 2 mM Tris–EDTA, 3 mM MgCl_2_, 5 mg/mL BSA, 50 μM phenylmethylsulfonyl fluoride; pH 7.4), [^35^S]-GTPγS (1,500–2,000 counts per minute), GDP (100 μM), and increasing concentrations (10^−9^-10^−4^) of a selective agonist for CB1 (methanandamide cat. M186 Sigma–Aldrich) or CB2 receptors (CB65 cat. 2663 Tocris). The total binding was estimated in the absence of the specific agonist, whereas non-specific binding was obtained in the presence of unlabeled GTPγS (100 μM). The specific binding was calculated by subtracting the non-specific binding from the total binding. The reaction was initiated by adding [^35^S]-GTPγS and was terminated by filtration of the samples through Whatman GF/B glass fiber filters and using a Brandel M-48 multifilter. Filters were washed three times with ice-cold 50 mM Tris–HCl buffer (pH 7.4) and 1 mg/mL BSA (pH 7.4) and dried. The filters were immersed in the Sigma Fluor™ scintillation liquid and 500 μL of 0.1% Triton X-100 (cat. 9002-93-1, Sigma–Aldrich). The radioactivity in the filters was estimated in counts per minute using a Beckman detector LS6000SC. The final values expressed in fmol/mg of protein activation were analyzed by non-linear regression to determine the receptor coupling efficiency or maximum stimulation (Emax) induced by the different agonists and its potency (pEC50). Each sample was evaluated by triplicate.

### Statistical Analysis

Student *t* test was used to evaluate the results obtained from the different experiments. Pearson correlation coefficients were calculated to determine the possible influence of clinical conditions on the experimental results. Values were expressed as the mean ± SEM. GraphPad Software Inc. (v.6.01 USA) was used for the statistical analysis.

## Results

### Identification of CB1 and CB2 Receptors in Human Microvessels by Immunofluorescence Staining

**Figure 1:**
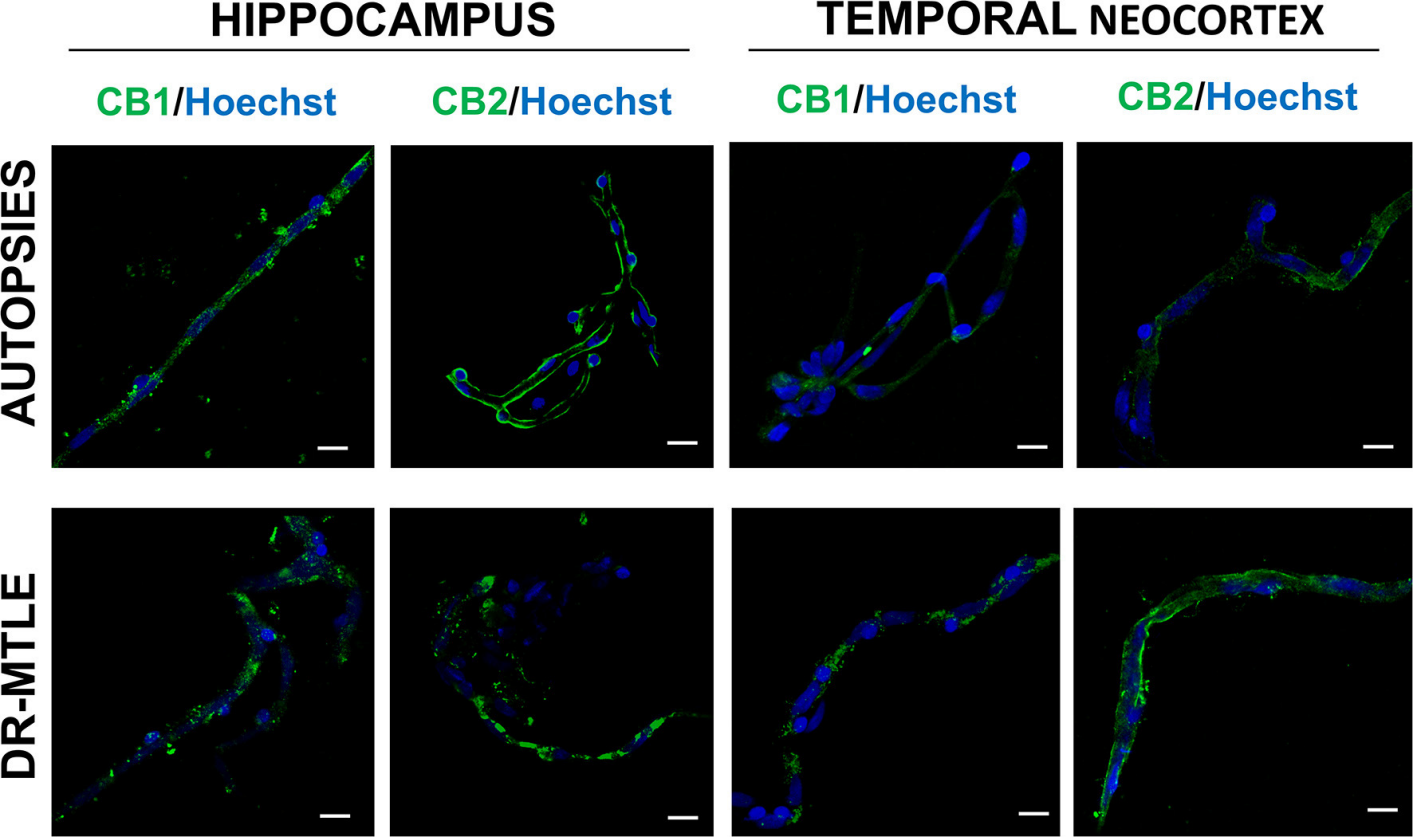
Immunofluorescence staining of CB1 and CB2 receptor proteins in microvessels isolated from the hippocampus and temporal neocortex of non-epileptic autopsies and patients with DR-MTLE. Cell nuclei were stained with Hoechst (in blue). In the group of patients with DR-MTLE, a low staining of CB1 and CB2 receptors can be observed in the hippocampus, whereas both receptors are highly expressed in the temporal neocortex. All scale bars are 20 μm.

**Figure 2:**
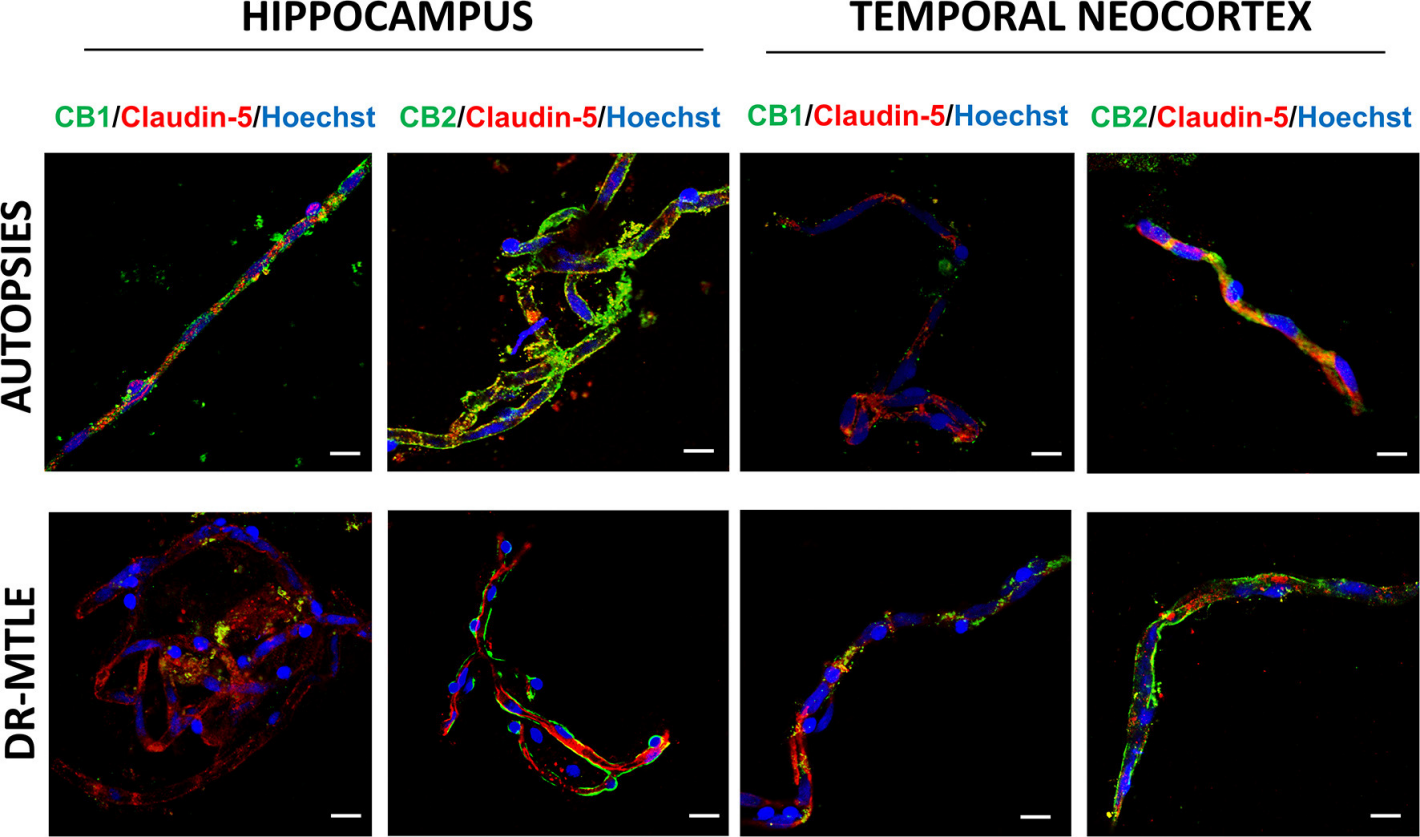
Double-labeling immunofluorescence staining of claudin-5 and CB1 or CB2 in MECs isolated from the hippocampus and temporal neocortex of non-epileptic autopsies and patients with DR-MTLE. Cell nuclei were stained with Hoechst (in blue). Notice the high claudin-5 staining colocalization with low (hippocampus) and high (temporal neocortex) CB1 and CB2 staining in MECs of patients with DR-MTLE. All scale bars are 20 μm.

**Figure 3:**
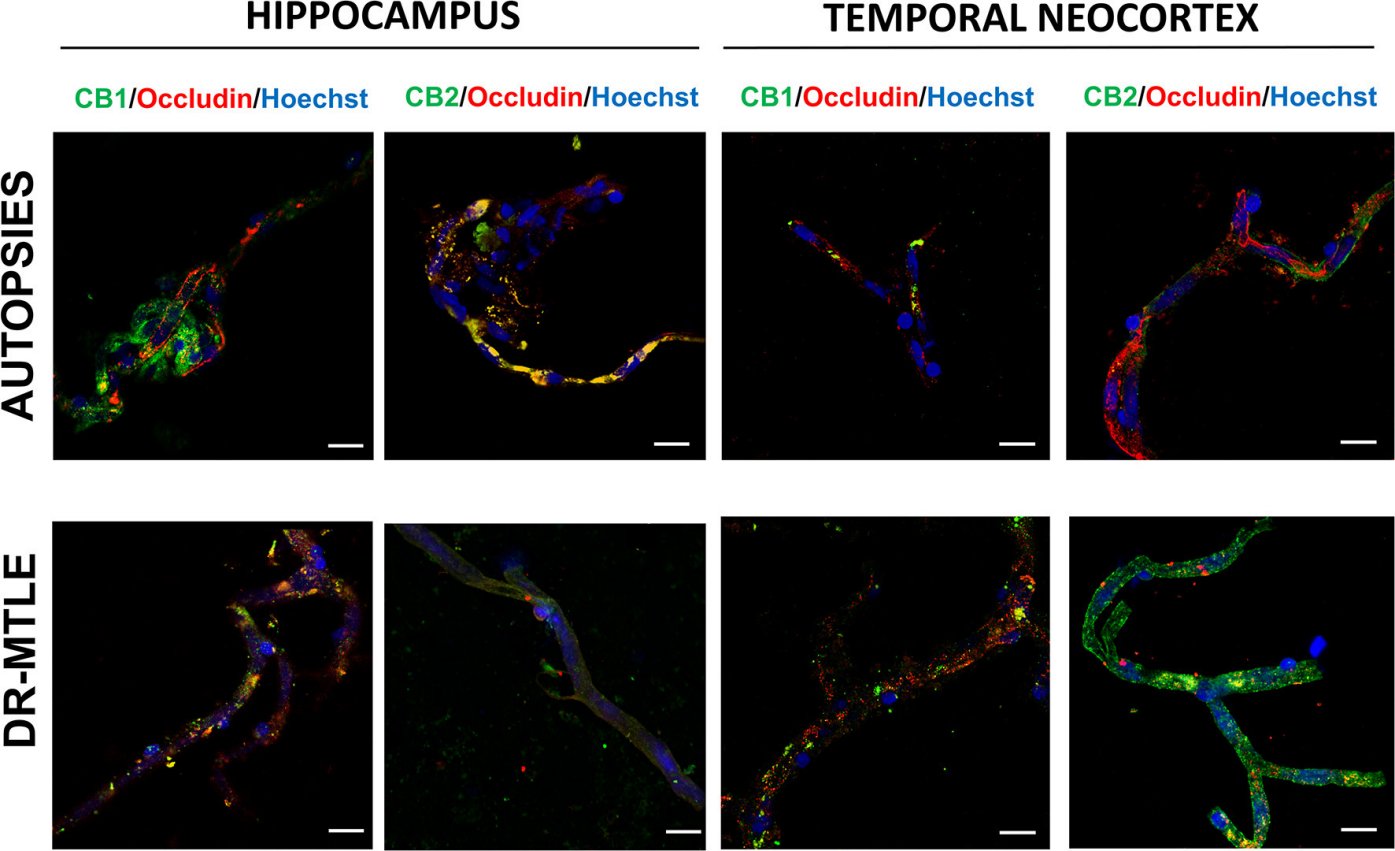
Double-labeling immunofluorescence staining of occludin and CB1 or CB2 in MECs isolated from the hippocampus and temporal neocortex of non-epileptic autopsies and patients with DR-MTLE. Cell nuclei were stained with Hoechst (in blue). Notice the low occludin staining colocalization with low (hippocampus) and high (temporal neocortex) CB1 and CB2 staining in MECs of patients with DR-MTLE. All scale bars are 20 μm.

**Figure 4:**
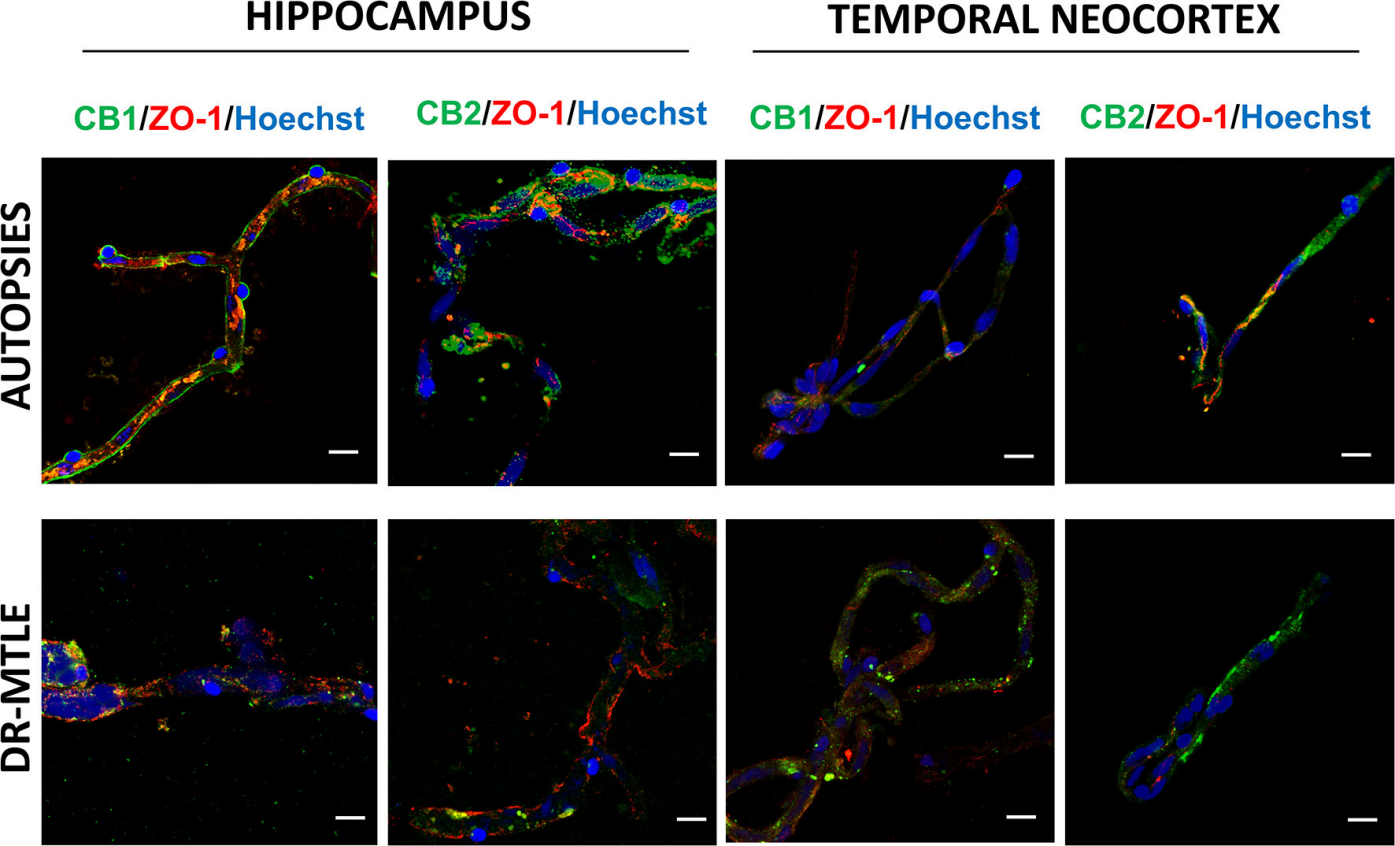
Double-labeling immunofluorescence staining of zonula occludens-1 (ZO-1) and CB1 or CB2 in MECs isolated from the hippocampus and temporal neocortex of non-epileptic autopsies and patients with DR-MTLE. Cell nuclei were stained with Hoechst (in blue). Notice the low ZO-1 staining colocalization with low (hippocampus) and high (temporal neocortex) CB1 and CB2 staining in MECs of patients with DR-MTLE. All scale bars are 20 μm.

**Figure 5:**
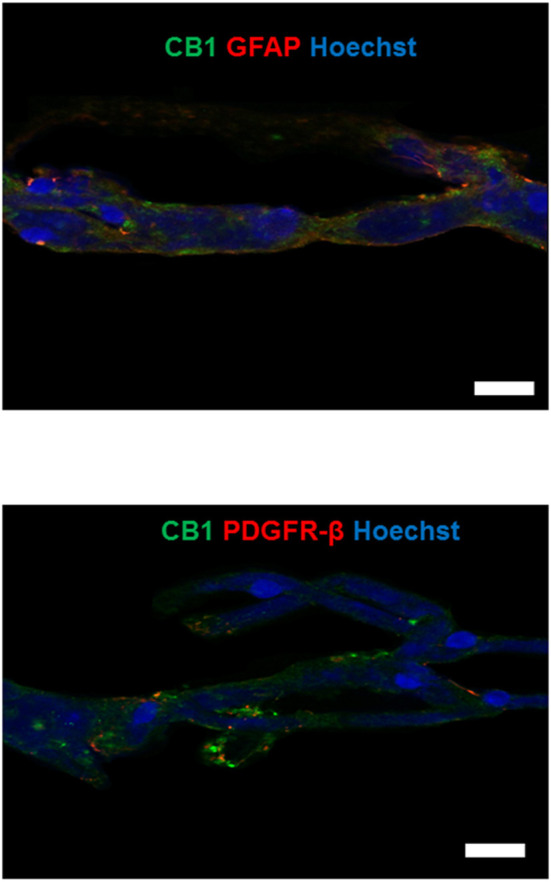
Upper panel: double-labeling immunofluorescence staining of CB1 receptors and GFAP in microvessels isolated from the hippocampus of a non-epileptic autopsy. Lower panel: double-labeling immunofluorescence staining of CB1 receptors and PDGFR-β in microvessels isolated from the temporal neocortex of a patient with DR-MTLE. Cell nuclei were stained with Hoechst (in blue). All scale bars are 20 μm.

The immunofluorescence images and their Z-stack analysis confirmed that receptors CB1 and CB2 proteins were expressed in the brain microvessels obtained from patients with DR-MTLE and non-epileptic autopsies (Figure 1; Supplementary Videos 1–4). Immunofluorescent double-labeling showed that CB1 and CB2 receptors colocalize with claudin-5, occludin, and ZO-1 proteins (Figures 2–4). Immunofluorescent experiments also displayed that CB1 and CB2 receptors colocalize with GFAP and PDGFR-β (Figure 5). These results support that both CB receptors are expressed in the human isolated microvessels fragments consisting of MECs with tight junction proteins, astrocyte end feet, and pericytes. Quantification of the immunofluorescence staining images was not carried out as the procedure used was qualitative and based on observations by a histologist.

### Protein Expression Levels of CB1 and CB2 Receptors and Functional Coupling to Gαi/o Proteins in Brain Microvessels of Autopsies

**Figure 6:**
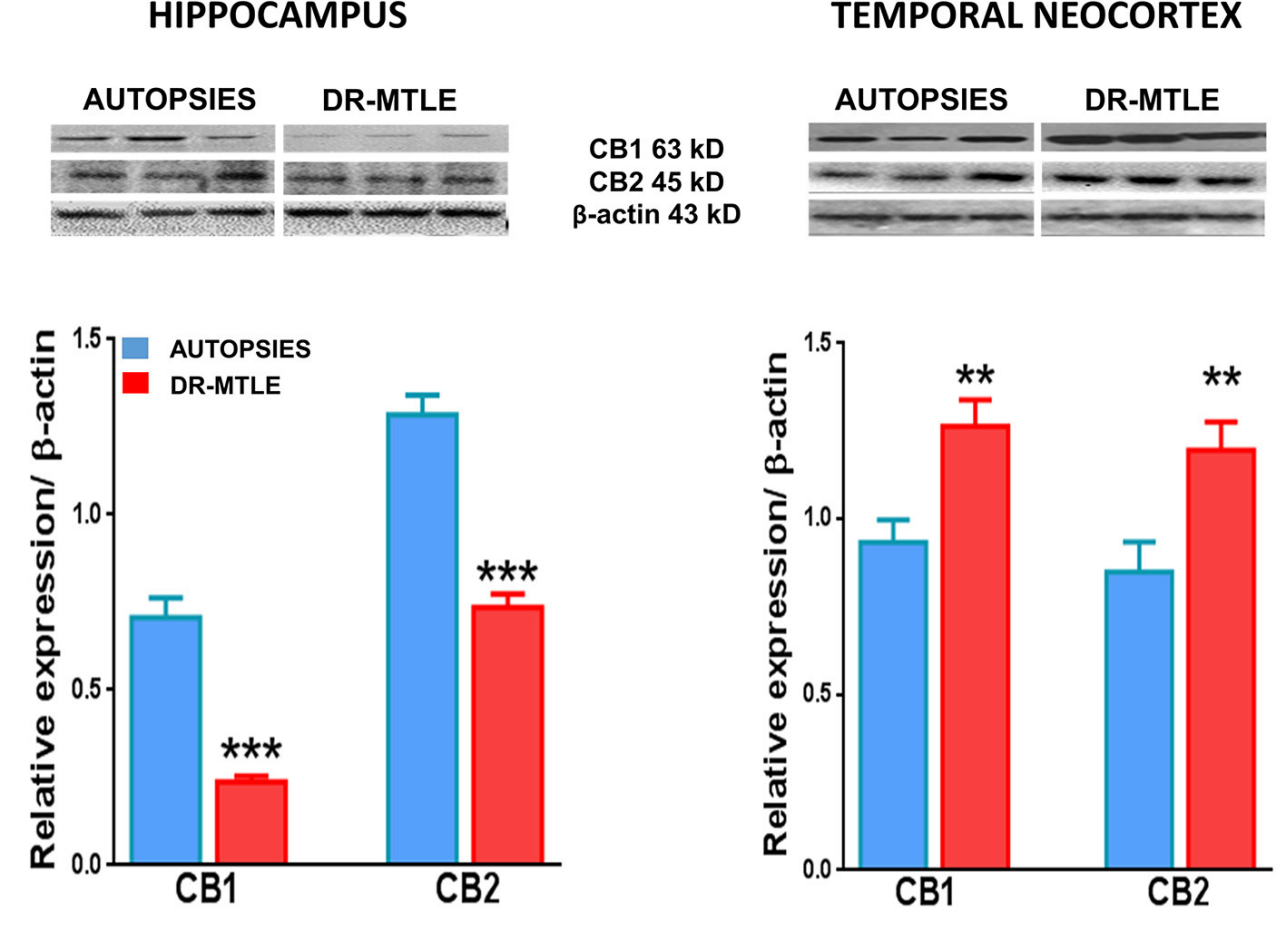
CB1 and CB2 protein expression in human microvessels obtained from non-epileptic autopsies and patients with DR-MTLE. Upper panels: Representative Western blots of CB1 and CB2 receptor proteins in microvessels isolated from the hippocampus and temporal neocortex of non-epileptic autopsies and patients with DR-MTLE. Lower panels: Representation of the densitometric analysis. The results are expressed as a percentage of the optical density (O.D.) ratio of CB1 and CB2-immunostained bands to those of β-actin. Data are presented as mean ± standard error. **p < 0.01, ***p < 0.001.

**Figure 7:**
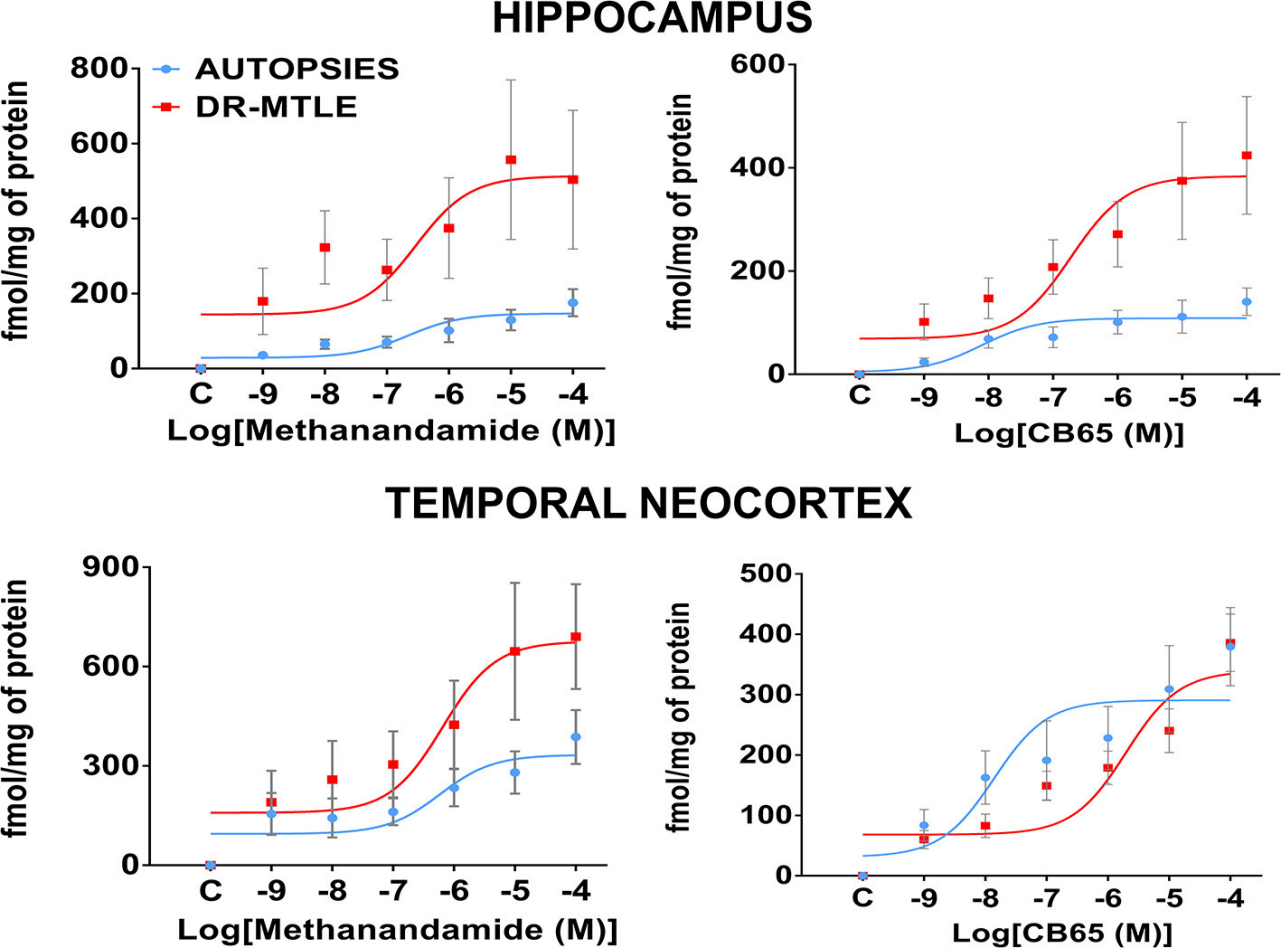
Gαi/o protein activation induced by agonists of CB1 and CB2 receptors in the human microvessels. Specific [^35^S]-GTPγS binding to cell membranes obtained from microvessels of the hippocampus and the temporal neocortex of non-epileptic autopsies and patients with DR-MTLE as a function of increasing concentrations of methanandamide (CB1 agonist) and CB65 (CB2 agonist). Each point represents the mean ± SE of the individual percentage stimulation over the basal values. Values are expressed as fmol/mg of protein.

Western blot experiments showed that CB1 and CB2 receptors were expressed in brain microvessels of non-epileptic autopsies. Protein expression of CB1 and CB2 receptors was as follows: hippocampus, 0.62 ± 0.09 and 1.25 ± 0.08, respectively; temporal neocortex, 0.93 ± 0.07 and 0.84 ± 0.04, respectively (Figure 6). The binding of [^35^S]-GTPγS induced by the exposure of specific agonists to CB1 and CB2 receptors in microvessels of the hippocampus and temporal neocortex of autopsies was concentration-dependent (Figure 7). Emax and pEC_50_ values attained are indicated in Table 3. No significant correlations were detected between the results obtained and the age of subjects or the postmortem interval (Table 4). According to these results, data obtained from the autopsy group were considered as the control condition.

**Table 3:** Maximum stimulation (Emax) and potency (pEC_50_) values for methanandamide (CB1 agonist) and CB65 (CB2 agonist) inducing [^35^S]GTPγS binding in microvessels of the hippocampus and temporal neocortex of non-epileptic autopsies and patients with DR-MTLE.

**Receptor, brain area, and group**	**Emax fmol/mg prot**	***p < ***	**pEC_**50**_**	***p < ***
CB1 hippocampus autopsies	146 ± 11	0.0008^*^	−6.65 ± 0.34	0.8687
CB1 hippocampus DR-MTLE	513 ± 93		−6.52 ± 0.7	
CB2 hippocampus autopsies	108 ± 10	0.0001^*^	−8.1 ± 0.37	0.035^*^
CB2 hippocampus DR-MTLE	384 ± 49		−6.7 ± 0.49	
CB1 neocortex autopsies	333 ± 45	0.006^*^	−6.23 ± 0.46	0.9459
CB1 neocortex DR-MTLE	676 ± 105		−6.18 ± 0.5	
CB2 neocortex autopsies	290.9 ± 29	0.2639	−7.86 ± 0.39	0.0002^*^
CB2 neocortex DR-MTLE	339.7 ± 30		−5.7 ± 0.26	

*Emax, maximum stimulation; pEC50, −log of half-effective concentration (potency); DR-MTLE, drug-resistant mesial temporal lobe epilepsy*.

* *p < 0.05*.

**Table 4:** Correlations between clinical data and results obtained from the evaluation of CB1 and CB2 receptors.

**Clinical data**	**CB1 receptor**						**CB2 receptor**					
	**Protein expression**		**Emax**		**pEC**_****50****_		**Protein expression**		**Emax**		**pEC**_****50****_	
	**Hipp**	**Cx**	**Hipp**	**Cx**	**Hipp**	**Cx**	**Hipp**	**Cx**	**Hipp**	**Cx**	**Hipp**	**Cx**
**Patients**												
Age (years)	−0.145	0.280	−0.026	−0.355	0.887^***^	−0.200	−0.033	−0.451	0.136	0.083	−0.210	−0.615
Age of seizure onset (years)	0.0452	−0.309	−0.406	−0.277	−0.070	0.204	0.275	0.127	−0.499	−0.377	0.040	0.037
Duration of epilepsy (years)	−0.123	0.224	0.191	−0.136	0.651^*^	−0.399	−0.273	−0.422	0.379	0.330	−0.075	−0.473
Frequency of Seizures (per month)	0.287	−0.051	−0.079	0.166	−0.445	0.115	−0.106	0.608	−0.130	−0.781^**^	0.278	0.633^*^
No. of ASD before surgery	0.103	0.644^*^	0.531	−0.188	−0.349	0.167	−0.643^*^	−0.265	0.318	0.064	0.092	0.231
**Autopsies**												
Age (years)	0.351	0.542	−0.178	−0.325	0.357	−0.297	−0.329	−0.428	−0.085	0.134	0.235	−0.447
PMI (h)	0.101	0.120	−0.274	0.409	0.472	0.370	−0.144	−0.129	0.426	−0.488	0.066	0.598

*ASD, antiseizure drugs; Emax, maximum stimulation; Hipp, hippocampus; Cx, cortex; pEC50, −log of half-effective concentration (potency); PMI, postmortem interval. Values represent Pearson correlation coefficient value (r)*.

* *p < 0.05*,

** *p < 0.01*,

*** *p < 0.001*.

### DR-MTLE Modifies the CB1 and CB2 Protein Expression and Functional Coupling to Gαi/o Proteins in Human Microvessels

Concerning the hippocampal microvessels of patients with DR-MTLE, Western blot experiments showed lower protein expression of CB1 and CB2 receptors (66 and 43%, respectively; *p* < 0.001) (Figure 6). This effect was associated with lower potency (pEC_50_) for CB2 receptors (*p* < 0.035) revealed by the binding assay. Despite this condition, the activation of CB1 and CB2 receptors resulted in a higher Gαi/o protein activation efficiency (Emax) (CB1, 251%, *p* < 0.0008 vs. autopsies; CB2, 255%, *p* < 0.0001 vs. autopsies) (Figure 7; Table 3).

The correlation analysis showed that CB1 receptors with the highest potency were found in the hippocampal microvessels of the oldest patients (*r* = 0.887, *p* < 0.001 vs. autopsies) and patients with the longest duration of the illness (*r* = 0.6509, *p* < 0.02 vs. autopsies) (Table 4).

Concerning the microvessels obtained from the temporal neocortex of patients with DR-MTLE, the Western blot evaluation showed high expression of CB1 receptors (35%, *p* < 0.01 vs. autopsies) (Figure 6). This effect was associated with higher efficiency for CB1-induced Gαi/o protein activation (103%, *p* < 0.006 vs. autopsies) revealed by the binding assay (Figure 7). Concerning CB2 receptors, protein overexpression was found with Western blot experiments (41%, *p* < 0.01 vs. autopsies). Despite this effect, the efficiency of CB2-induced Gαi/o protein activation was similar to autopsies (*p* < 0.2639 vs. autopsies), but with lower potency (*p* < 0.0004 vs. autopsies) (Figure 7; Table 3).

Opposite changes were found when hippocampus (diminished expression of CB1 and CB2) and temporal neocortex (increased expression of CB1 and CB2) of patients with DR-MTLE were compared. However, the comparison of the Gαi/o protein activation efficiency induced by the activation of CB1 and CB2 receptors in the epileptic hippocampus (513 ± 93 and 384 ± 49 fmol/mg protein, respectively) was similar when compared with the propagation area (temporal neocortex, 676 ± 105 and 339.7 ± 30 fmol/mg protein, respectively) (Table 3). According to this information, the Gαi/o protein activation efficiency in the hippocampus was similar to the temporal neocortex of patients with DR-MTLE.

Correlation analysis indicated that cortical microvessels of patients with DR-MTLE with the highest seizure frequency showed CB2 receptors with the lowest efficiency (*r* = −0.7807, *p* < 0.0077) and the highest potency (*r* = 0.6327, *p* < 0.0496) to induce Gαi/o protein activation. Correlation analysis also revealed that a highest number of antiseizure drugs received before the epilepsy surgery correlated with the highest protein expression of CB1 in the temporal neocortex and the lowest protein expression of CB2 receptors in the hippocampus (Table 4).

## Discussion

The results indicated that cerebral microvessels of patients with DR-MTLE and non-epileptic autopsies expressed CB1 and CB2 receptors, and their occupation by specific agonists induced the activation of Gαi/o proteins.

In the present study, we used an optimized protocol that allowed isolating microvessels from frozen brain tissue. The isolated microvessels contained MECs, pericytes (PDGFR-β), and astrocytes end feet (GFAP) (Castañeda-Cabral et al., 2020a; present study). Pericytes and astrocytes are also a source of CB1 and CB2 receptors (Benyó et al., 2016). Then, we cannot discard the influence of these cell elements in the results obtained.

The criteria we used to include autopsy tissue as control were according to the quality of mRNA of each sample. In addition, β-actin was used as a loading control for the Western blot experiments. The expression of mRNA and β-actin was always within the range of detection. We also investigated the correlation of the values obtained from the different experiments with the postmortem interval and the age of the subjects to determine their possible influence on the experimental results. According to our analysis, the postmortem interval or age of autopsies did not influence the results obtained from autopsies. However, we cannot completely confirm that the controls did not have neurodegenerative disorders. This situation represents a limitation of the present study.

Microvessels from the hippocampus and temporal neocortex of patients with DR-MTLE showed a higher efficiency to induce Gαi/o protein activation as a consequence of CB1 agonist exposure when compared to non-epileptic autopsies. In addition, CB1 receptors presented higher potency in the hippocampus of the patients with a longer duration of epilepsy. These findings suggest that the neurotransmission mediated by CB1 receptors is higher in the BBB of patients with DR-MTLE. This effect was detected in the hippocampus despite lower protein expression, suggesting an adaptive change in the proximal downstream signaling mediated by these receptors.

Binding assays revealed a higher efficiency of CB2 receptor-induced Gαi/o protein activation in microvessels of epileptic hippocampus regardless of low protein expression. These results indicated that CB2 receptors are more efficiently coupled to signal transduction mechanisms in the hippocampal BBB of patients with DR-MTLE. The functional coupling of CB2 receptors to Gαi/o proteins in the hippocampal and cortical BBB of patients with DR-MTLE was found to be lower than the potency parameter in the control autopsies. However, correlation analysis indicated that the cortical microvessels of patients with the highest seizure frequency showed the lowest efficiency but the highest potency for CB2 receptor-induced Gαi/o protein activation. These results indicated that the potency of the functional coupling of CB2 receptors might augment as a response to high seizure frequency.

Overall, these results suggest a differential role for CB1 and CB2 receptors in the hippocampal and cortical BBB of patients with DR-MTLE. Indeed, opposite changes were detected when hippocampus (diminished expression of CB1 and CB2) and temporal neocortex (increased expression of CB1 and CB2) were compared. This situation can be associated to clinical conditions. Concerning this issue, we found that the highest number of antiseizure drugs received before the surgery correlated with the lowest protein expression of CB2 receptors in the hippocampus and the highest protein expression of CB1 receptors in the temporal neocortex. However, the Gαi/o protein activation efficiency induced by the activation of CB1 and CB2 receptors in the hippocampus was similar when compared with temporal neocortex. Then, we can conclude that the Gαi/o protein activation efficiency in the epileptogenic area (hippocampus) was similar to the propagation zone (temporal neocortex) despite opposite changes in protein expression. Further studies are essential to determine the influence of clinical factors in the expression and function of CB1 and CB2 receptors in patients with epilepsy.

Some studies support the protecting role of CB1 and CB2 receptors in the integrity of BBB (Lu et al., 2008; Zhang et al., 2011; Ramirez et al., 2012). Indeed, the activation of CB1 and CB2 receptors by 2-arachidonoylglycerol, an endocannabinoid, contributes to maintaining the integrity of the BBB following a brain insult (Piro et al., 2018). On the other hand, microvessels of patients with DR-MTLE overexpress VEGF-A and its receptor VEGFR-2 (Castañeda-Cabral et al., 2020a). These changes may facilitate angiogenesis (Marchi and Lerner-Natoli, 2013), neuroinflammation, and increased BBB permeability (Gorter et al., 2015; Baruah et al., 2020). Activation of cannabinoid receptors is considered a strategy to attenuate VEGF signaling and chronic inflammation and thereby diminishing neoangiogenesis (Schley et al., 2009; Staiano et al., 2016; Sathyapalan et al., 2017). However, the high signal transduction efficiency mediated by the activation of cannabinoid receptors (present study) does not avoid BBB impairment associated with epilepsy (van Vliet et al., 2014; Broekaart et al., 2018). The lack of protective effects mediated by CB1 and CB2 receptors in the microvasculature of patients with DR-MTLE can be related to low tissue levels of endocannabinoids such as 2-arachidonoylglycerol (Rocha et al., 2020).

Nanocarriers have been designed to overcome the BBB and deliver drugs to the brain parenchyma (Naz and Siddique, 2020). However, BBB represents a valuable target in the treatment of neurodegenerative disorders. The present study indicates that brain microvessels with high signal transmission mediated by CB1 and CB2 receptors may represent a novel therapeutic target to preserve the integrity of BBB of patients with DR-MTLE.

On the other hand, experimental evidence suggests that the activation of CB1 and CB2 receptors could induce neurotoxicity, a condition that may depend on the chronicity of the disorder (Di Marzo, 2008; Fowler et al., 2010; Vendel and de Lange, 2014). Neurodegenerative disorders studies also indicate that the activation of CB1 and CB2 receptors may augment the migration of immune cells (Miller and Stella, 2008) and thus facilitate oxidative stress (Mukhopadhyay et al., 2010), inflammation, and the disruption of BBB (Persidsky et al., 2006). Studies indicate that activation of CB1 and CB2 receptors augments leukocyte accumulation, inflammation, and neovascularization (Guabiraba et al., 2013). Our results revealed that patients with DR-MTLE showed a high signal transduction efficiency mediated by the activation of CB1 and CB2 receptors, a condition that may facilitate inflammation and neovascularization in the hippocampus and temporal neocortex. Further studies are essential to determine if the high-efficiency coupling to the signal transduction mechanisms mediated by CB1 and CB2 receptors facilitates the impairment of the BBB in patients with DR-MTLE.

## Data Availability Statement

The raw data supporting the conclusions of this article will be made available by the authors, without undue reservation.

## Ethics Statement

The studies involving human participants were reviewed and approved by Instituto Nacional de Neurologia and Neurocirugia and Centro de Investigación y de Estudios Avanzados. The patients/participants provided their written informed consent to participate in this study.

## Author Contributions

LR designed the study and organized the manuscript. MN-L carried out the Western blot and binding [^35^S]-GTPγS assay experiments. JC-C carried out the immunofluorescence experiments. MV-D, FW, and MD designed the procedure for isolating microvessels from the frozen human brain. VS-V participated in the Western blot experiments. SO-S participated in the analysis of immunofluorescence experiments. RG-G obtained and evaluated the autopsy samples. IM-J identified and assessed the patients with epilepsy. MA-V did the neurosurgery of patients. FC-C participated in the [^35^S]-GTPγS assay experiments. All authors contributed to the article and approved the submitted version.

## Conflict of Interest

The authors declare that the research was conducted in the absence of any commercial or financial relationships that could be construed as a potential conflict of interest.
