# Supplementary material for: Drug-Resistant Temporal Lobe Epilepsy Alters the Expression and Functional Coupling to Gαi/o Proteins of CB1 and CB2 Receptors in the Microvasculature of the Human Brain
Source: Front Behav Neurosci. 2021 Jan 20;14:611780. doi: 10.3389/fnbeh.2020.611780 (PMC7854549; doi:10.3389/fnbeh.2020.611780)
Supplement: Supplementary file 1 [file Data_Sheet_1.PDF]

Supplementary Table 1. Antibodies used for the western blot and immunofluorescence experiments.

**Antibodies for Western-Blot**			
**Primary antibody**	**Dilution**	**Secondary antibodies**	**Dilution**
CB1 receptor: 2F9: sc-293419 (against amino acids 1-110 of RCB1 of human origin) Santa Cruz Biotechnology	1:1000	m-IgGκ BP-HRP:sc-516102 anti-mouse Santa Cruz Biotechnology	1:10000
CB2 receptor: 3C7: sc-293188 (against amino acids 302-360 of RCB2 of human origin) Santa Cruz Biotechnology	1:1000	m-IgGκ BP-HRP:sc-516102 anti-mouse Santa Cruz Biotechnology	1:10000
Occludin: sc-133256 Biorbyt	1:500	IgG PI-1000 anti-rabbit. Vector	1:10000
Claudin-5: sc 28670 Santa Cruz Biotechnology	1:500	IgG PI-1000 anti-rabbit Vector	1:10000
Zonula occludens-1: sc-10804 Santa Cruz Biotechnology	1:500	IgG PI-1000 anti-rabbit Vector	1:10000
β-actin: sc-47778 Santa Cruz Biotechnology	1:5000	m-IgGκ BP-HRP:sc-516102 anti-mouse Santa Cruz Biotechnology	1:10000
**Antibodies for immunofluorescence**			
CB1 receptor: 2F9: sc-293419 (against amino acids 1-110 of RCB1 of human origin) Santa Cruz Biotechnology	1:100	Alexa Fluor® 488 anti-mouse	1:200
CB2 receptor: 3C7: sc-293188 (against amino acids 302-360 of RCB2 of human origin) Santa Cruz Biotechnology	1:100	Alexa Fluor® 488 anti-mouse	1:200
Occludin: sc-133256 Biorbyt	1:200	Alexa Fluor® 546 anti-rabbit	1:200
Claudin-5: sc 28670 Santa Cruz Biotechnology	1:200	Alexa Fluor® 546 anti-rabbit	1:200
Zonula occludens -1: sc-10804 Santa Cruz Biotechnology	1:200	Alexa Fluor® 546 anti-rabbit	1:200
GFAP: 20334 Dako	1:200	Alexa Fluor® 546 anti-rabbit	1:200
PDGFR-β: sc-19995 Santa Cruz Biotechnology	1:200	Alexa Fluor® 546 anti-rabbit	1:200
